# Transcriptional regulatory network discovery via multiple method integration: application to *e. coli *K12

**DOI:** 10.1186/1748-7188-2-2

**Published:** 2007-03-30

**Authors:** Jingjun Sun, Kagan Tuncay, Alaa Abi Haidar, Lisa Ensman, Frank Stanley, Michael Trelinski, Peter Ortoleva

**Affiliations:** 1 Center for Cell and Virus Theory, Chemistry Building, Indiana University, Bloomington, IN 47405, USA

## Abstract

Transcriptional regulatory network (TRN) discovery from one method (e.g. microarray analysis, gene ontology, phylogenic similarity) does not seem feasible due to lack of sufficient information, resulting in the construction of spurious or incomplete TRNs. We develop a methodology, TRND, that integrates a preliminary TRN, microarray data, gene ontology and phylogenic similarity to accurately discover TRNs and apply the method to *E. coli *K12. The approach can easily be extended to include other methodologies. Although gene ontology and phylogenic similarity have been used in the context of gene-gene networks, we show that more information can be extracted when gene-gene scores are transformed to gene-transcription factor (TF) scores using a preliminary TRN. This seems to be preferable over the construction of gene-gene interaction networks in light of the observed fact that gene expression and activity of a TF made of a component encoded by that gene is often out of phase. TRND multi-method integration is found to be facilitated by the use of a Bayesian framework for each method derived from its individual scoring measure and a training set of gene/TF regulatory interactions. The TRNs we construct are in better agreement with microarray data. The number of gene/TF interactions we discover is actually double that of existing networks.

## Background

The growing number of gene expression datasets and availability of hundreds of bacterial genomes accelerated the quest for the construction of bacterial transcriptional regulatory networks (TRNs). In most prokaryotic genes, transcription initiation is controlled by DNA sequence elements recognized by RNA polymerase. The activity of RNA polymerase (RP) is regulated through interaction with transcription factors (TFs) which alter the binding affinity of RP. Discovery of TRNs advances our understanding of mechanisms of cellular processes and responses, and is of particular importance in biotechnical applications and identifying the nature of diseases from a genome-wide perspective. Our objective in this work is to develop a robust methodology to use known TRN information as a training set and augment it by discovering new gene/TF interactions using a variety of approaches integrated via an objective Bayesian scheme.

We apply the methodology to *E. coli *as it is believed to have the most well understood TRN; therefore it serves as an excellent test case. However, out of roughly 4300 genes and around 300 predicted TFs [1], the current *E. coli *TRN includes only 984 genes and 144 TFs. Hence, it is clear that we only know a fraction of the network. According to Babu and Teichmann three-quarters of the TFs are two-domain proteins, i.e., DNA-binding domain and regulatory domain (mostly for small molecules), showing the importance of TFs in adapting to environmental conditions [1]. Like most biological interaction networks, the *E. coli *network seems to follow a power law (scale free) distribution, suggesting that TRNs tend to be connected among high-degree nodes and low-degree ones [2]. Another important property of TRNs is the statistically overrepresented network motifs. Shen-Orr et al. showed that the feed forward motif (two TFs co-regulating one gene and one TF regulating the other) is overrepresented by a factor of 8 in the known *E. coli *TRN [3]. These studies advance our understanding of design principles in bacterial TRNs. However, they do not have a direct impact on the construction of TRNs.

There have been numerous approaches to TRN inference from gene expression data. Most studies considered gene-gene networks rather than gene-TF networks. Among them are principal component analysis [4] and independent component analysis [5]. Network component analysis (NCA) is a TF-based methodology which differs from other techniques in that the structure of the gene regulatory network is assumed to be known [6]. Therefore, NCA's use is limited to cases in which the network is fairly well known and has strong structural limitations. In reality, only an incomplete and possibly biased TRN is available due to the limited spectrum of experimental conditions imposed. Gardner et al. proposed a methodology to construct the gene-gene control network structure for small networks using microarray data, limiting the number of interactions per gene [7]. We tested a similar approach for large networks and showed that even when there are just a few interactions per gene, there can be thousands of networks that can explain the same microarray data with essentially the same accuracy. Kyoda et al. developed a methodology that employs mutation experiments to arrive at the TRN [8]. However, it is questionable whether their approach can be applied to large TRNs. Liang et al. presented a methodology for Boolean networks and applied it to a small 50 gene system with at most 3 interactions per gene [9]. Boolean networks are an oversimplification of gene expression as they use a binary approximation (fully on or off) [10]. Cluster analysis is based on statistical techniques wherein correlations are sought between the responses of genes [11,12]. However the coordination can be extremely complex and circuitous, i.e. genes may be involved in a multi-branch feedback loop with several TFs made or activated/deactivated by the proteins they encode. These time-delayed, complex relationships are revealed by our methodology as it discovers and quantifies many of these feedback relationships. Although cluster analysis might suggest groups of genes that may be involved in related pathways, it is not an accurate methodology to suggest gene/TF interactions. D'haeseleer et al. applied clustering based on the correlation of microarray data [13].

**Figure 1:**
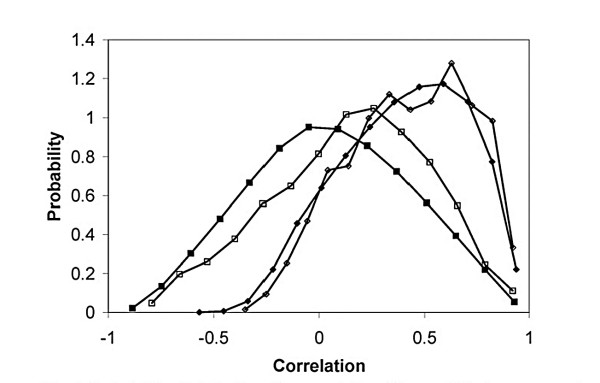
Probability distribution for correlation (Pearson) between a random pair and known gene/TF regulatory interaction for *E. coli*. Square markers refer to the dataset obtained from the U. of Oklahoma E. coli database. Diamond markers refer to the datasets obtained from the NIH omnibus service (GSE7, GSE8, GSE9; 65 datasets). The solid and hollow markers show the probability distribution for correlation between a random gene pair and known gene/TF regulatory interaction, respectively. As these probability distributions are indistinguishable, it does not seem feasible to construct the TRN using expression data alone. We also calculated probability distributions for mutual information which yielded similar findings.

To assess the feasibility of inferring gene-gene networks from expression data only, we used two independent gene expression data sets and a TRN for *E. coli *[14]. We calculated the linear correlation of genes that encode a TF and genes that are known to be regulated by the same TF. We also obtained correlation coefficients for all gene-gene pairs. Fig. 1 shows the probability of correlation between two randomly chosen genes and that for known pairs with similar known gene/TF interactions. Throughout the manuscript we compute probability densities. These probability density functions are normalized to have unit area although their value at any score can exceed unity (∫−∞∞p(x′)dx′=1
MathType@MTEF@5@5@+=feaafiart1ev1aaatCvAUfKttLearuWrP9MDH5MBPbIqV92AaeXatLxBI9gBaebbnrfifHhDYfgasaacH8akY=wiFfYdH8Gipec8Eeeu0xXdbba9frFj0=OqFfea0dXdd9vqai=hGuQ8kuc9pgc9s8qqaq=dirpe0xb9q8qiLsFr0=vr0=vr0dc8meaabaqaciaacaGaaeqabaqabeGadaaakeaadaWdXbqaaiabdchaWjabcIcaOiqbdIha4zaafaGaeiykaKIaemizaqMafmiEaGNbauaaaSqaaiabgkHiTiabg6HiLcqaaiabg6HiLcqdcqGHRiI8aOGaeyypa0JaeGymaedaaa@3C5A@). The actual probability can then be calculated by taking the integral of the function *p*(*x*) by the integration interval of the input variable *x*. The similarity of these distributions demonstrates that successful reconstruction of the network using expression data alone does not seem likely. Mutual information seems to have similar limitations [15]. However, this does not mean that correlation and mutual information-based methods are not able to discover interesting gene-gene relationships; rather their potential to infer gene/TF interactions is very limited. Therefore, the main assumption in constructing gene-gene networks, i.e. that the TF activity follows the expression of the encoding gene seems to be unreliable. We address this problem by constructing approximate TF activity profiles using a preliminary TRN as discussed below.

The difficulty with the above studies is the gap between the complexity of the network and the quantity of information in just one methodology. The solution is to use as much information as possible to rule out spurious networks. Segal et al. assumed that genes in the same pathway are activated together and their protein products often interact [16]. This led them to the use of protein-protein interaction information in their predictions. Brazma et al. studied the similarities of the upstream regions of genes that have a similar expression pattern [17]. A similar study was presented by Haverty et al. who used statistical methods for identifying overabundant TF binding motifs (from TRANSFAC and JASPER) and microarray data to infer the TRN [18]. Lee et al. presented a conceptual framework to integrate diverse functional genomics data (including expression data, gene-fusions, phylogenetic profiles, co-citation, and protein interaction data) and applied it to investigate gene-gene network in *Saccharomyces cerevisiae *[19]. The major difference between [19] and this work is that we are interested in constructing gene/TF networks rather than gene-gene networks.

Gene ontology (GO) and phylogenic similarity as approaches to functional module prediction have been explored by [20]. This work is based on the hypothesis that a pair of genes with high GO or phylogenic similarity score is likely in the same functional module (operon or regulon). In this study, we extend their work to include gene expression analysis, and focus on TRN construction. We show that GO and phylogenic similarity can be used to greatest advantage if they are based on a gene/TF interaction model.

## Methods

### Network definition

The TRN we seek to discover is a list of genes for each of which a set of TFs with up/down regulation is provided (*b*_*in *_= +1/-1 for gene *i *up/down regulated by TF *n*). The gene-gene regulation network often considered is implied as the components of each TF and the genes that encode them are also included in our TRNs. This TRN definition provides a unifying framework for all the individual TRN discovery methods we developed, as well as a methodology for the integration of multiple methods. We use multiple methodologies to suggest enhanced TRNs based on three hypotheses and a training set TRN to test them. The result of each methodology is weighed proportional to its success rate using the training set. This approach goes beyond studies that focus on gene-gene networks as it provides more detailed information (such as gene A is up regulated by TF B) that can be tested experimentally and used in medical and biotechnical applications. We demonstrate that methodologies such as gene ontology and phylogenic similarity provide better results when a preliminary set of gene/TF interactions is used instead of a training set of gene-gene data. A simple algorithm, described below, is used to calculate gene-TF scores from gene-gene similarity scores and a preliminary TRN. In addition, we use a novel approach to first approximate TF activity profiles using the preliminary TRN and gene expression data, and then using these TF activities to suggest additional gene/TF interactions via a gene-TF correlation scheme.

### From gene-gene scores to gene-TF scores

Two of the methodologies (GO and phylogeny) used in this study generate gene-gene similarity scores. As our interest is the discovery of TRNs as defined above, the question is how one can use the gene-gene similarity scores and the preliminary TRN to score gene/TF interactions. For a system of *N*_*gene *_genes, there are *N*_*gene *_× (*N*_*gene *_- 1)/2 gene-gene pairs. In order to find the score for gene A and TF B, we first seek all genes regulated by TF B in the preliminary TRN. Then we calculate the gene-gene similarity score for the gene of interest with each gene regulated by TF B. We assign the maximum of these scores to the gene A/TF B interaction. Although this appears to be a rough estimation of the gene-TF score, our computational experiments with gene-gene similarity based on gene ontology and phylogeny have shown that this score clearly distinguishes the probability distributions of the training and random sets of gene/TF interactions.

### Gene ontology analysis

In this analysis we use the biological process ontology developed by the Gene Ontology (GO) consortium [21,22], the GO annotations from EMBL-EBI [23] and hypothesize that the likelihood for a gene pair to be regulated in the same manner increases with the similarity of their GO description. GO analysis was proposed by [20] who applied it to find functional modules in *E. coli*. However, here a training set of gene/TF interactions is used instead of a gene-gene pair-based one. In particular, we use a preliminary *E. coli *TRN and transform the gene-gene scores to gene-TF scores. Each GO is structured as a directed acyclic graph. The GO similarity score between two gene products is based on the number of shared ancestors. As a gene product might be assigned with multiple GO terms, we seek the maximum similarity score between all possible combinations. Let gene *i *and gene *j *be assigned *h*_*i *_and *h*_*j *_GO terms, respectively. Then the GO similarity for the gene (*i*, *j*) pair is taken to be the maximum number of shared ancestors for all combinations of the *h*_*i *_and *h*_*j*_.

### Phylogenic similarity analysis

Phylogenic similarity analysis, also proposed by [20], is based on the hypothesis that a pair of genes with large phylogenic similarity score is likely in the same functional operon, regulon or pathway. Our implementation differs in that we suggest that if two genes have high phylogenic similarity score, then they would be regulated in the same manner by the same set of TFs. Based on this hypothesis we extend the preliminary TRN.

Our approach is to calculate phylogenic similarity for gene-gene pairs follows the methodology proposed by [20] (referred to as 'likelihood of neighboring profiles' in their work). In this analysis all bacteria sequence information is downloaded from [24] and all preliminary gene/TF interactions are from [14]. Once we have phylogenic similarity scores for all gene pairs, we calculate the gene/TF scores based on the methodology described in the From Gene-Gene Scores to Gene/TF Scores Section.

#### Calculation of the phylogenic similarity

We first construct a vector for each gene in *E. coli*, the dimension of the vector being the number of genomes used in the analysis (in this study 229). We applied BLASTP to identify probable orthologous genes of a target genome in 229 reference genomes. The most significant BLASTP hit from each reference species was considered the true ortholog of the target species if the expectation value was less than 1.0e-10 [25]. If there is an orthologous gene in the *i*^*th *^genome, then the *i*^*th *^entry in this vector is assigned the order of the orthologous gene in the *i*^*th *^genome. If an orthologous gene does not exist in the *i*^*th *^genome, then this entry is taken to be *0*. Once such a vector for each *E. coli *gene is constructed, we compute a phylogenic similarity measure for each gene pair. Given two vectors *X*_*i *_= [*x*_*i*1_, *x*_*i*2_,...,*x*_*i*229_] for gene *i *and similarly *X*_*j *_for gene *j*, we use the following phylogenic similarity measure for a gene pair:

SijPHY=−∑k=1229log⁡[P(xik,xjk)].     (1)
 MathType@MTEF@5@5@+=feaafiart1ev1aaatCvAUfKttLearuWrP9MDH5MBPbIqV92AaeXatLxBI9gBaebbnrfifHhDYfgasaacH8akY=wiFfYdH8Gipec8Eeeu0xXdbba9frFj0=OqFfea0dXdd9vqai=hGuQ8kuc9pgc9s8qqaq=dirpe0xb9q8qiLsFr0=vr0=vr0dc8meaabaqaciaacaGaaeqabaqabeGadaaakeaacqWGtbWudaqhaaWcbaGaemyAaKMaemOAaOgabaGaemiuaaLaemisaGKaemywaKfaaOGaeyypa0JaeyOeI0YaaabCaeaacyGGSbaBcqGGVbWBcqGGNbWzcqGGBbWwcqWGqbaucqGGOaakcqWG4baEdaWgaaWcbaGaemyAaKMaem4AaSgabeaakiabcYcaSiabdIha4naaBaaaleaacqWGQbGAcqWGRbWAaeqaaOGaeiykaKIaeiyxa0faleaacqWGRbWAcqGH9aqpcqaIXaqmaeaacqaIYaGmcqaIYaGmcqaI5aqoa0GaeyyeIuoakiabc6caUiaaxMaacaWLjaWaaeWaaeaacqaIXaqmaiaawIcacaGLPaaaaaa@568D@

Here *P*(*x*_*ik*_, *x*_*jk*_), the likelihood of genes *i *and *j*, is calculated from

=(1−pik)(1−pjk)ifxik=0 andxjk=0P(xik,xjk)=pik(1−pjk)ifxik≠0 andxjk=0=(1−pik)pjkifxik=0 andxjk≠0=pikpjkd(xik,xjk)(2Nk−d(xik,xjk)−1)Nk(Nk−1)ifxik≠0 andxjk≠0     (2)
 MathType@MTEF@5@5@+=feaafiart1ev1aaatCvAUfKttLearuWrP9MDH5MBPbIqV92AaeXatLxBI9gBaebbnrfifHhDYfgasaacH8akY=wiFfYdH8Gipec8Eeeu0xXdbba9frFj0=OqFfea0dXdd9vqai=hGuQ8kuc9pgc9s8qqaq=dirpe0xb9q8qiLsFr0=vr0=vr0dc8meaabaqaciaacaGaaeqabaqabeGadaaakeaafaqaaaabdaaaaeaaaeaacqGH9aqpcqGGOaakcqaIXaqmcqGHsislcqWGWbaCdaWgaaWcbaGaemyAaKMaem4AaSgabeaakiabcMcaPiabcIcaOiabigdaXiabgkHiTiabdchaWnaaBaaaleaacqWGQbGAcqWGRbWAaeqaaOGaeiykaKcabaacbaGae8xAaKMae8NzayMae8hiaaIaemiEaG3aaSbaaSqaaiabdMgaPjabdUgaRbqabaGccqGH9aqpcqaIWaamcqqGGaaicqWFHbqycqWFUbGBcqWFKbazcqWFGaaicqWG4baEdaWgaaWcbaGaemOAaOMaem4AaSgabeaakiabg2da9iabicdaWaqaaiabdcfaqjabcIcaOiabdIha4naaBaaaleaacqWGPbqAcqWGRbWAaeqaaOGaeiilaWIaemiEaG3aaSbaaSqaaiabdQgaQjabdUgaRbqabaGccqGGPaqkaeaacqGH9aqpcqWGWbaCdaWgaaWcbaGaemyAaKMaem4AaSgabeaakiabcIcaOiabigdaXiabgkHiTiabdchaWnaaBaaaleaacqWGQbGAcqWGRbWAaeqaaOGaeiykaKcabaGae8xAaKMae8NzayMae8hiaaIaemiEaG3aaSbaaSqaaiabdMgaPjabdUgaRbqabaGccqGHGjsUcqaIWaamcqqGGaaicqWFHbqycqWFUbGBcqWFKbazcqWFGaaicqWG4baEdaWgaaWcbaGaemOAaOMaem4AaSgabeaakiabg2da9iabicdaWaqaaaqaaiabg2da9iabcIcaOiabigdaXiabgkHiTiabdchaWnaaBaaaleaacqWGPbqAcqWGRbWAaeqaaOGaeiykaKIaemiCaa3aaSbaaSqaaiabdQgaQjabdUgaRbqabaaakeaacqWFPbqAcqWFMbGzcqWFGaaicqWG4baEdaWgaaWcbaGaemyAaKMaem4AaSgabeaakiabg2da9iabicdaWiabbccaGiab=fgaHjab=5gaUjab=rgaKjab=bcaGiabdIha4naaBaaaleaacqWGQbGAcqWGRbWAaeqaaOGaeyiyIKRaeGimaadabaaabaGaeyypa0JaemiCaa3aaSbaaSqaaiabdMgaPjabdUgaRbqabaGccqWGWbaCdaWgaaWcbaGaemOAaOMaem4AaSgabeaakmaalaaabaGaemizaqMaeiikaGIaemiEaG3aaSbaaSqaaiabdMgaPjabdUgaRbqabaGccqGGSaalcqWG4baEdaWgaaWcbaGaemOAaOMaem4AaSgabeaakiabcMcaPiabcIcaOiabikdaYiabd6eaonaaBaaaleaacqWGRbWAaeqaaOGaeyOeI0IaemizaqMaeiikaGIaemiEaG3aaSbaaSqaaiabdMgaPjabdUgaRbqabaGccqGGSaalcqWG4baEdaWgaaWcbaGaemOAaOMaem4AaSgabeaakiabcMcaPiabgkHiTiabigdaXiabcMcaPaqaaiabd6eaonaaBaaaleaacqWGRbWAaeqaaOGaeiikaGIaemOta40aaSbaaSqaaiabdUgaRbqabaGccqGHsislcqaIXaqmcqGGPaqkaaaabaGae8xAaKMae8NzayMae8hiaaIaemiEaG3aaSbaaSqaaiabdMgaPjabdUgaRbqabaGccqGHGjsUcqaIWaamcqqGGaaicqWFHbqycqWFUbGBcqWFKbazcqWFGaaicqWG4baEdaWgaaWcbaGaemOAaOMaem4AaSgabeaakiabgcMi5kabicdaWaaacaWLjaGaaCzcamaabmaabaGaeGOmaidacaGLOaGaayzkaaaaaa@F695@

where

*p*_*ik *_is the probability that gene *i *is present in genome *k*.

*N*_*k *_is the total number of genes in reference genome *k*

*d*(*x*_*ik*_, *x*_*jk*_) = *abs*(*x*_*ik *_- *x*_*jk*_).

To calculate *p*_*ik*_, we grouped 229 reference genomes into subgroups based on information gathered from [26,27] (see Table 1). It is assumed that *p*_*ik *_is identical within each subgroup for each gene. Then *p*_*ik *_is taken to be the ratio of number of genomes that has an orthologous gene to the total number of genomes in the subgroup.

**Table 1:** The list of bacteria used in the phylogenic similarity analysis.

**Subgroup**	**Bacteria**
Actinobacteria	Bifidobacterium longum NCC2705, Corynebacterium diphtheriae NCTC 13129, Corynebacterium efficiens YS-314, Corynebacterium glutamicum ATCC13032, Corynebacterium glutamicum ATCC 13032, Leifsonia xyli subsp. xyli str. CTCB07, Mycobacterium avium subsp. paratuberculosis str. k10, Mycobacterium bovis AF2122/97, Mycobacterium leprae TN, Mycobacterium tuberculosis H37Rv, Mycobacterium tuberculosis CDC1551, Nocardia farcinica IFM 10152, Propionibacterium acnes KPA171202, Streptomyces avermitilis MA-4680, Streptomyces coelicolor A3(2), Symbiobacterium thermophilum IAM 14863, Tropheryma whipplei TW08/27, Tropheryma whipplei str. Twist

Aquificae	Aquifex aeolicus VF5

Bacteroidetes	Bacteroides fragilis YCH46, Bacteroides fragilis NCTC 9343, Bacteroides thetaiotaomicron VPI-5482, Porphyromonas gingivalis W83

Cyanobacteria	Prochlorococcus marinus subsp. marinus str. CCMP1375, Prochlorococcus marinus str. MIT 9313

Chlamydiae	Chlamydophila abortus S26/3, Chlamydia muridarum Nigg, Chlamydia trachomatis D/UW-3/CX, Chlamydophila caviae GPIC, Chlamydophila pneumoniae AR39, Chlamydophila pneumoniae CWL029, Chlamydophila pneumoniae J138, Chlamydophila pneumoniae TW-183, Parachlamydia sp. UWE25

Chlorobi	Chlorobium tepidum TLS

Chloroflexi	Dehalococcoides ethenogenes 195

Crenarchaeota	Aeropyrum pernix K1, Pyrobaculum aerophilum str. IM2, Sulfolobus solfataricus P2, Sulfolobus tokodaii str. 7

Cyanobacteria	Gloeobacter violaceus PCC 7421, Nostoc sp. PCC 7120, Prochlorococcus marinus subsp. pastoris str. CCMP1986, Synechococcus elongatus PCC 6301, Synechococcus sp. WH 8102, Synechocystis sp. PCC 6803, Thermosynechococcus elongatus BP-1

Deinococcus-Thermus	Deinococcus radiodurans R1, Thermus thermophilus HB27, Thermus thermophilus HB8

Euryarchaeota	Archaeoglobus fulgidus DSM 4304, Haloarcula marismortui ATCC 43049, Halobacterium sp. NRC-1, Methanothermobacter thermautotrophicus str.Delta H, Methanocaldococcus jannaschii DSM 2661, Methanococcus maripaludis S2, Methanopyrus kandleri AV19, Methanosarcina acetivorans C2A, Methanosarcina mazei Go1, Picrophilus torridus DSM 9790, Pyrococcus abyssi GE5, Pyrococcus furiosus DSM 3638, Pyrococcus horikoshii OT3, Thermococcus kodakaraensis KOD1, Thermoplasma acidophilum DSM 1728, Thermoplasma volcanium GSS1

Firmicutes	Bacillus anthracis str. Ames, Bacillus anthracis str. 'Ames Ancestor', Bacillus anthracis str. Sterne, Bacillus cereus ATCC 14579, Bacillus cereus ATCC 10987, Bacillus cereus ZK, Bacillus clausii KSM-K16, Bacillus halodurans C-125, Bacillus licheniformis ATCC 14580, Bacillus subtilis subsp. subtilis str. 168, Bacillus thuringiensis serovar konkukian str. 97-27, Clostridium acetobutylicum ATCC 824, Clostridium perfringens str. 13, Clostridium tetani E88, Enterococcus faecalis V583, Geobacillus kaustophilus HTA426, Lactobacillus acidophilus NCFM, Lactobacillus johnsonii NCC 533, Lactobacillus plantarum WCFS1, Lactococcus lactis subsp. lactis Il1403, Listeria innocua Clip11262, Listeria monocytogenes EGD-e, Listeria monocytogenes str. 4b F2365, Mesoplasma florum L1, Mycoplasma gallisepticum R, Mycoplasma genitalium G-37, Mycoplasma hyopneumoniae 232, Mycoplasmamobile 163K, Mycoplasma mycoides subsp. mycoides SC str. PG1, Mycoplasma penetrans HF-2, Mycoplasma pneumoniae M129, Mycoplasma pulmonis UAB CTIP, Oceanobacillus iheyensis HTE831, Onion yellows phytoplasma OY-M, Staphylococcus aureus subsp. aureus COL, Staphylococcus aureus subsp. aureus MW2, Staphylococcus aureus subsp. aureus Mu50, Staphylococcus aureus subsp. aureus N315, Staphylococcus aureus subsp. aureus MRSA252, Staphylococcus aureus subsp. aureus MSSA476, Staphylococcus epidermidis ATCC 12228, Staphylococcus epidermidis RP62A, Streptococcus agalactiae 2603V/R, Streptococcus agalactiae NEM316, Streptococcus mutans UA159, Streptococcus pneumoniae R6, Streptococcus pneumoniaeTIGR4, Streptococcus pyogenes M1 GAS, Streptococcus pyogenes MGAS10394, Streptococcus pyogenes MGAS315, Streptococcus pyogenes MGAS8232, Streptococcus pyogenes SSI-1, Streptococcus thermophilus CNRZ1066, Streptococcus thermophilus LMG 18311, Thermoanaerobacter tengcongensis MB4, Ureaplasma parvum serovar 3 str. ATCC 700970

Fusobacteria	Fusobacterium nucleatum subsp. nucleatum ATCC 25586

Nanoarchaeota	Nanoarchaeum equitans Kin4-M

Planctomycetes	Rhodopirellula baltica SH 1

Proteobacteria	Acinetobacter sp. ADP1, Agrobacterium tumefaciens str. C58, Agrobacterium tumefaciens str. C58, Anaplasma marginale str. St. Maries, Azoarcus sp. EbN1, Bartonella henselae str. Houston-1, Bartonella quintana str. Toulouse, Bdellovibrio bacteriovorus HD100, Candidatus Blochmannia floridanus, Bordetella bronchiseptica RB50, Bordetella parapertussis 12822, Bordetella pertussis Tohama I, Bradyrhizobium japonicum USDA 110, Brucella abortus biovar 1 str. 9–941, Brucella melitensis 16M, Brucella suis 1330, Buchnera aphidicola str. Bp (Baizongia pistaciae), Buchnera aphidicola str. Sg (Schizaphis graminum), Buchnera aphidicola str. APS (Acyrthosiphon pisum), Burkholderia mallei ATCC 23344, Burkholderia pseudomallei K96243, Campylobacter jejuni subsp. jejuni NCTC 11168, Campylobacter jejuni RM1221, Caulobacter crescentus CB15, Chromobacterium violaceum ATCC 12472, Coxiella burnetii RSA 493, Desulfotalea psychrophila LSv54, Desulfovibrio vulgaris subsp. vulgaris str. Hildenborough, Ehrlichia ruminantium str. Gardel, Ehrlichia ruminantium str. Welgevonden, Ehrlichia ruminantium str. Welgevonden, Erwinia carotovora subsp. atroseptica SCRI1043, Escherichia coli CFT073, Escherichia coli K12, Escherichia coli O157:H7 EDL933, Escherichia coli O157:H7, Francisella tularensis subsp. tularensis Schu 4, Gluconobacter oxydans 621H, Geobacter sulfurreducens PCA, Haemophilus ducreyi 35000HP, Haemophilus influenzae Rd KW20, Helicobacter hepaticus ATCC 51449, Helicobacter pylori 26695, Helicobacter pylori J99, Idiomarina loihiensis L2TR, Legionella pneumophila str. Lens, Legionella pneumophila str. Paris, Legionella pneumophila subsp. pneumophila str. Philadelphia 1, Mannheimia succiniciproducens MBEL55E, Mesorhizobium loti MAFF303099, Methylococcus capsulatus str. Bath, Neisseria gonorrhoeae FA 1090, Neisseria meningitidis MC58, Neisseria meningitidis Z2491, Nitrosomonas europaea ATCC 19718, Pasteurella multocida subsp.multocida str. Pm70, Photobacterium profundum SS9, Photorhabdus luminescens subsp. laumondii TTO1, Pseudomonas aeruginosa PAO1, Pseudomonas putida KT2440, Pseudomonas syringae pv. syringae B728a, Pseudomonas syringae pv. tomato str. DC3000, Ralstonia solanacearum GMI1000, Rhodopseudomonas palustris CGA009, Rickettsia conorii str. Malish 7, Rickettsia prowazekii str. Madrid E, Rickettsia typhi str. Wilmington, Salmonella enterica subsp. enterica serovar Choleraesuis str. SC-B67, Salmonella enterica subsp. enterica serovar Paratyphi A str. ATCC 9150, Salmonella enterica subsp. enterica serovar Typhi str. CT18, Salmonella enterica subsp. enterica serovar Typhi Ty2, Salmonella typhimurium LT2, Shewanella oneidensis MR-1, Shigella flexneri 2a str. 301, Silicibacter pomeroyi DSS-3, Sinorhizobium meliloti 1021, Shigella flexneri 2a str. 2457T, Vibrio cholerae O1 biovar eltor str. N16961, Vibrio fischeri ES114, Vibrio parahaemolyticus RIMD 2210633, Vibriovulnificus CMCP6, Vibrio vulnificus YJ016, Wigglesworthia glossinidia endosymbiont of Glossina brevipalpis, Wolbachia endosymbiont strain TRS of Brugia malayi, Wolbachia endosymbiont of Drosophila melanogaster, Wolinella succinogenes DSM 1740, Xanthomonas campestris pv. campestris str. ATCC 33913, Xylella fastidiosa 9a5c, Xanthomonas axonopodis pv. citri str. 306, Xanthomonas campestris pv. campestris str. 8004, Xanthomonas oryzae pv. oryzae KACC10331, Xylella fastidiosa Temecula1, Yersinia pestis biovar Medievalis str. 91001, Yersinia pestis CO92, Yersinia pestis KIM, Yersinia pseudotuberculosis IP 32953, Zymomonas mobilis subsp. mobilis ZM4

Spirochaetes	Borrelia burgdorferi B31, Borrelia garinii PBi chromosome linear, Leptospira interrogans serovar Copenhageni str. Fiocruz L1-130, Leptospira interrogans serovar Lai str. 56601, Treponema denticola ATCC 35405, Treponema pallidum subsp. pallidum str. Nichols

Thermotogae	Thermotoga maritima MSB8

### Microarray analysis

Kinetic cell models hold great promise for predicting cell behavior [28-32]. Unfortunately there is a lack of information about many of the rate and equilibrium constants for the reaction and transport processes involved [33,34]. Simultaneously calibrating all the reaction/transport rate parameters and discovering the gene/TF interaction network structure from available data does not appear to be feasible. Therefore, instead of using a kinetic approach as a basis of TRN construction, we have developed FTF (Fast Transcription Factor analyzer) for network construction via (1) TF activity estimation, (2) statistical arguments, and (3) a preliminary TRN. Once a reliable TRN is obtained using FTF, it can then be used to calibrate the rate and equilibrium constants that appear in transcription/translation kinetic models. An example of such an approach is available at [35].

FTF was designed based on the following notions:

• a method based on TFs has the advantage that microarray noise, and errors in preliminary TRN, can be overcome by statistics – i.e. the regulation of many genes by a given TF;

• due to data uncertainty, there is not usually enough information content in many single-gene responses to unambiguously determine the effect of all TFs regulating it; and

• TRN discovery requires many automated trials of possible networks, so the algorithm must be efficient.

#### Calculation of TF activities using FTF

The essential equation on which FTF is based was arrived at empirically after extensive numerical experimentation with synthetic data. In this way we actually know the TRN, TF activities, and the nature of noise added to the expression data, and thereby could quantitatively assess the accuracy of FTF predictions. FTF is based on the following ansatz:

Tnr−Tns=∑i=1NgeneH(mir−mis)binΨin,     (3)
 MathType@MTEF@5@5@+=feaafiart1ev1aaatCvAUfKttLearuWrP9MDH5MBPbIqV92AaeXatLxBI9gBaebbnrfifHhDYfgasaacH8akY=wiFfYdH8Gipec8Eeeu0xXdbba9frFj0=OqFfea0dXdd9vqai=hGuQ8kuc9pgc9s8qqaq=dirpe0xb9q8qiLsFr0=vr0=vr0dc8meaabaqaciaacaGaaeqabaqabeGadaaakeaacqWGubavdaqhaaWcbaGaemOBa4gabaGaemOCaihaaOGaeyOeI0Iaemivaq1aa0baaSqaaiabd6gaUbqaaiabdohaZbaakiabg2da9maaqahabaGaemisaGKaeiikaGIaemyBa02aa0baaSqaaiabdMgaPbqaaiabdkhaYbaakiabgkHiTiabd2gaTnaaDaaaleaacqWGPbqAaeaacqWGZbWCaaGccqGGPaqkcqWGIbGydaWgaaWcbaGaemyAaKMaemOBa4gabeaakiabfI6aznaaBaaaleaacqWGPbqAcqWGUbGBaeqaaaqaaiabdMgaPjabg2da9iabigdaXaqaaiabd6eaonaaBaaameaacqWGNbWzcqWGLbqzcqWGUbGBcqWGLbqzaeqaaaqdcqGHris5aOGaeiilaWIaaCzcaiaaxMaadaqadaqaaiabiodaZaGaayjkaiaawMcaaaaa@5D38@

where Tnr
 MathType@MTEF@5@5@+=feaafiart1ev1aaatCvAUfKttLearuWrP9MDH5MBPbIqV92AaeXatLxBI9gBaebbnrfifHhDYfgasaacH8akY=wiFfYdH8Gipec8Eeeu0xXdbba9frFj0=OqFfea0dXdd9vqai=hGuQ8kuc9pgc9s8qqaq=dirpe0xb9q8qiLsFr0=vr0=vr0dc8meaabaqaciaacaGaaeqabaqabeGadaaakeaacqWGubavdaqhaaWcbaGaemOBa4gabaGaemOCaihaaaaa@30DC@ = activity of TF *n *at condition or time *r*, mir
 MathType@MTEF@5@5@+=feaafiart1ev1aaatCvAUfKttLearuWrP9MDH5MBPbIqV92AaeXatLxBI9gBaebbnrfifHhDYfgasaacH8akY=wiFfYdH8Gipec8Eeeu0xXdbba9frFj0=OqFfea0dXdd9vqai=hGuQ8kuc9pgc9s8qqaq=dirpe0xb9q8qiLsFr0=vr0=vr0dc8meaabaqaciaacaGaaeqabaqabeGadaaakeaacqWGTbqBdaqhaaWcbaGaemyAaKgabaGaemOCaihaaaaa@3104@ = microarray response of gene *i *at condition *r*, *b*_*in *_= TRN (*b*_*in *_= +1/-1for gene *i *up/down regulated by TF *n*, *b*_*in *_= 0 for no regulation), *H*(*x*) = ± 1 for *x *> or < 0, = *0 *for *x *= *0*, and Ψ_*in *_= 2Li
 MathType@MTEF@5@5@+=feaafiart1ev1aaatCvAUfKttLearuWrP9MDH5MBPbIqV92AaeXatLxBI9gBaebbnrfifHhDYfgasaacH8akY=wiFfYdH8Gipec8Eeeu0xXdbba9frFj0=OqFfea0dXdd9vqai=hGuQ8kuc9pgc9s8qqaq=dirpe0xb9q8qiLsFr0=vr0=vr0dc8meaabaqaciaacaGaaeqabaqabeGadaaakeaacqaIYaGmdaahaaWcbeqaaiabdYeamnaaBaaameaacqWGPbqAaeqaaaaaaaa@3074@/(*M*_*n*_(2Li
 MathType@MTEF@5@5@+=feaafiart1ev1aaatCvAUfKttLearuWrP9MDH5MBPbIqV92AaeXatLxBI9gBaebbnrfifHhDYfgasaacH8akY=wiFfYdH8Gipec8Eeeu0xXdbba9frFj0=OqFfea0dXdd9vqai=hGuQ8kuc9pgc9s8qqaq=dirpe0xb9q8qiLsFr0=vr0=vr0dc8meaabaqaciaacaGaaeqabaqabeGadaaakeaacqaIYaGmdaahaaWcbeqaaiabdYeamnaaBaaameaacqWGPbqAaeqaaaaaaaa@3074@ - 1)) for *L*_*i *_= number of TFs controlling gene *i *and *M*_*n *_= number of genes TF *n *regulates. If there are *N*_*expression *_times or conditions, then eq. (1) constitutes *N*_*expression *_× (*N*_*expression *_-1)/2 equations for the *N*_*expression *_activities Tnr
 MathType@MTEF@5@5@+=feaafiart1ev1aaatCvAUfKttLearuWrP9MDH5MBPbIqV92AaeXatLxBI9gBaebbnrfifHhDYfgasaacH8akY=wiFfYdH8Gipec8Eeeu0xXdbba9frFj0=OqFfea0dXdd9vqai=hGuQ8kuc9pgc9s8qqaq=dirpe0xb9q8qiLsFr0=vr0=vr0dc8meaabaqaciaacaGaaeqabaqabeGadaaakeaacqWGubavdaqhaaWcbaGaemOBa4gabaGaemOCaihaaaaa@30DC@ for each of the TFs. Therefore, the problem is overdetermined. In our approach the problem is solved via normal equations, i.e. using a least square approach so that all the expression data is utilized and thereby statistics can help to overcome data uncertainty.

Once TF activities are calculated in this manner, the linear (Pearson) correlation is calculated for all possible gene-TF pairs. This serves as a score used to construct probability distributions for the training set (known gene/TF interactions) and random set (all possible gene/TF pairs). Comparison of these probability distributions gives an idea about the fitness of the preliminary TRN and expression data, and to which degree we can rely on the predictions of FTF. If the preliminary TRN is too small or of poor quality, or if there are too few expression datasets, the training versus random set probability distributions are difficult to distinguish. The scores can also be used to rank genes that are more likely to have expression data which is inconsistent with the preliminary TRN.

**Figure 2:**
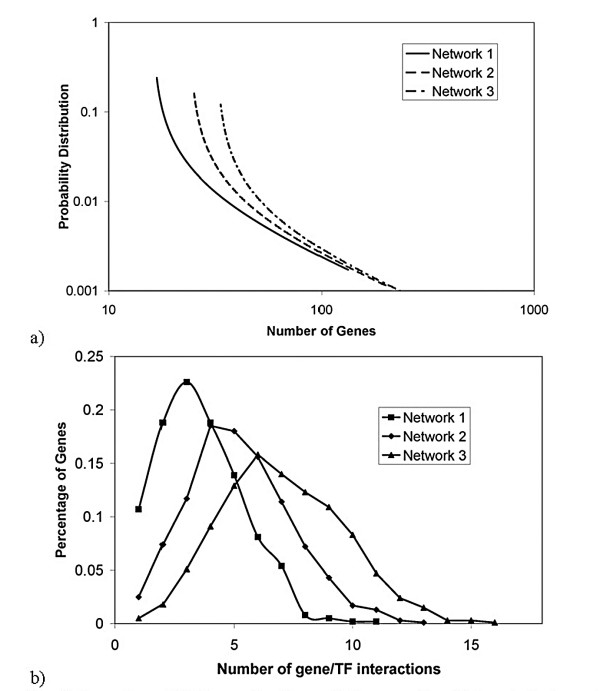
Properties of TRNs used in the synthetic examples. Networks that consist of 1000 genes and 100 TFs are generated using the probability distribution for the number of genes regulated by a given TF shown in (a). The corresponding probability distribution for the number of regulators per gene is shown in (b). The average number of regulators per gene is 3.62, 5.22, and 7.02 for Networks 1, 2 and 3, respectively. Equal likelihood is chosen for up versus down regulation.

To test FTF we generated a TRN that consists of 1000 genes and 100 TFs. The properties of the TRN are shown in Fig. 2. The synthetic expression data was generated by assumed random TF activities. Expression data for gene *i *was generated using mir=∑n=1NTFQinbinTnr
 MathType@MTEF@5@5@+=feaafiart1ev1aaatCvAUfKttLearuWrP9MDH5MBPbIqV92AaeXatLxBI9gBaebbnrfifHhDYfgasaacH8akY=wiFfYdH8Gipec8Eeeu0xXdbba9frFj0=OqFfea0dXdd9vqai=hGuQ8kuc9pgc9s8qqaq=dirpe0xb9q8qiLsFr0=vr0=vr0dc8meaabaqaciaacaGaaeqabaqabeGadaaakeaacqWGTbqBdaqhaaWcbaGaemyAaKgabaGaemOCaihaaOGaeyypa0ZaaabCaeaacqWGrbqudaWgaaWcbaGaemyAaKMaemOBa4gabeaakiabdkgaInaaBaaaleaacqWGPbqAcqWGUbGBaeqaaOGaemivaq1aa0baaSqaaiabd6gaUbqaaiabdkhaYbaaaeaacqWGUbGBcqGH9aqpcqaIXaqmaeaacqWGobGtdaWgaaadbaGaemivaqLaemOrayeabeaaa0GaeyyeIuoaaaa@47D2@. Here, mir
 MathType@MTEF@5@5@+=feaafiart1ev1aaatCvAUfKttLearuWrP9MDH5MBPbIqV92AaeXatLxBI9gBaebbnrfifHhDYfgasaacH8akY=wiFfYdH8Gipec8Eeeu0xXdbba9frFj0=OqFfea0dXdd9vqai=hGuQ8kuc9pgc9s8qqaq=dirpe0xb9q8qiLsFr0=vr0=vr0dc8meaabaqaciaacaGaaeqabaqabeGadaaakeaacqWGTbqBdaqhaaWcbaGaemyAaKgabaGaemOCaihaaaaa@3104@ is the expression level of gene *i *at experiment *r*, Tnr
 MathType@MTEF@5@5@+=feaafiart1ev1aaatCvAUfKttLearuWrP9MDH5MBPbIqV92AaeXatLxBI9gBaebbnrfifHhDYfgasaacH8akY=wiFfYdH8Gipec8Eeeu0xXdbba9frFj0=OqFfea0dXdd9vqai=hGuQ8kuc9pgc9s8qqaq=dirpe0xb9q8qiLsFr0=vr0=vr0dc8meaabaqaciaacaGaaeqabaqabeGadaaakeaacqWGubavdaqhaaWcbaGaemOBa4gabaGaemOCaihaaaaa@30DC@ is the activity of TF *n *at experiment *r*, *N*_*TF *_is the number of TFs, and *Q*_*in *_is a measure of the binding affinity of TF *n *and gene *i*.

**Figure 3:**
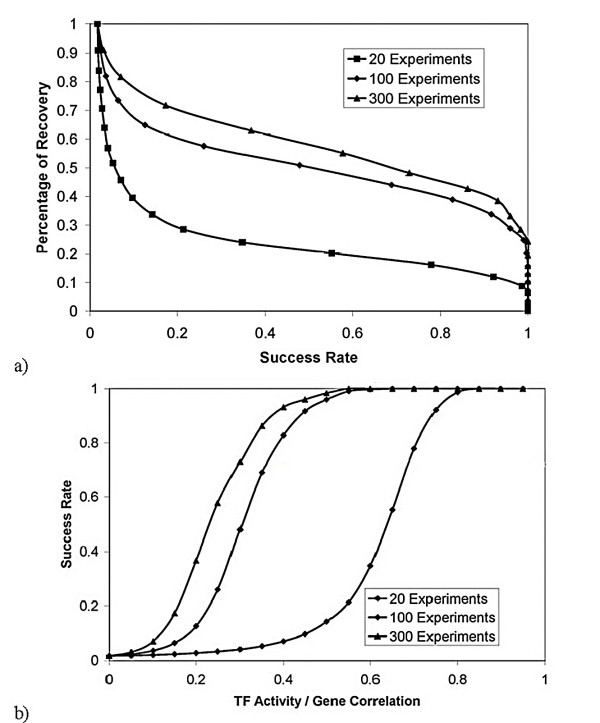
Reconstruction of TRNs. We have used the Network 1 of Fig. 2 and generated synthetic expression data. Then, we eliminated 50% of the network (randomly), and used FTF to reconstruct the deleted network. Fig. a) shows the percentage of the deleted network recovered as a function of success rate, a measure of the likelihood that an interaction is correct, as estimated from the training set (known interactions). As the number of microarray experiments increases, a higher percentage of the network can be reconstructed. However, full reconstruction requires too many experiments. Fig. b) shows success rate as a function of the absolute value of the linear correlation between the constructed TF activity profiles and gene expression data.

**Figure 4:**
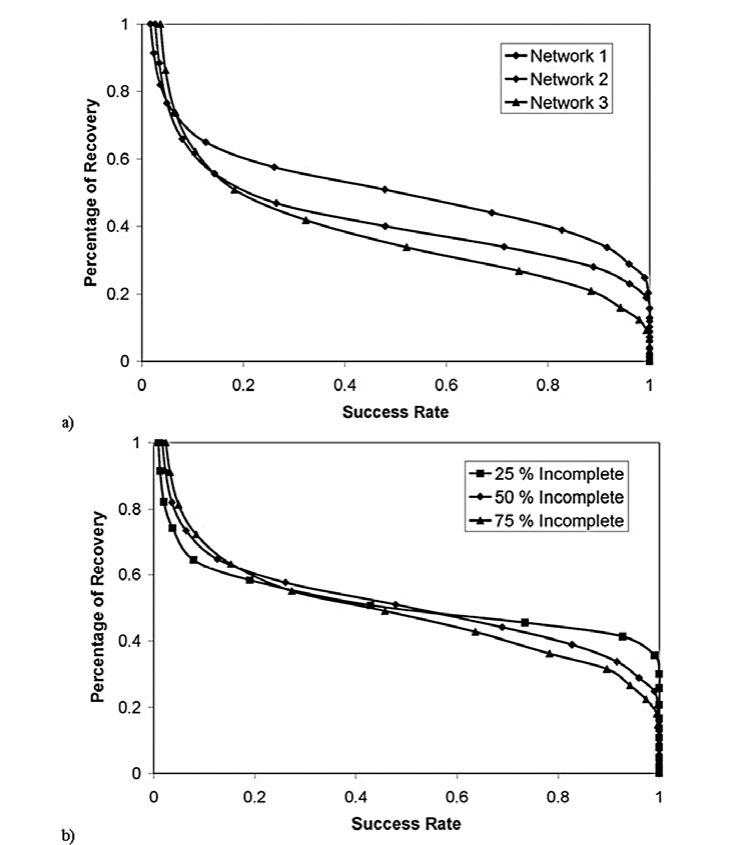
Effect of TRN properties. We used Networks 1, 2 and 2 of Fig. 2 to generate 100 synthetic expression data sets, and eliminated 50% of the gene/TF interactions in the TRN. Shown is the percentage of the deleted network recovered as a function of success rate. As the number interactions increases, the percentage of the network that can be recovered decreases. b) Same as a) except we used Network 1 and eliminated 25%, 50%, and 75% of the network. As expected, higher percentage of the deleted network is recoverable when a more complete network is known.

To construct a synthetic TRN, for each TF we assigned *u*_*n *_= *c*_1 _+ c2e−c3z
 MathType@MTEF@5@5@+=feaafiart1ev1aaatCvAUfKttLearuWrP9MDH5MBPbIqV92AaeXatLxBI9gBaebbnrfifHhDYfgasaacH8akY=wiFfYdH8Gipec8Eeeu0xXdbba9frFj0=OqFfea0dXdd9vqai=hGuQ8kuc9pgc9s8qqaq=dirpe0xb9q8qiLsFr0=vr0=vr0dc8meaabaqaciaacaGaaeqabaqabeGadaaakeaacqWGJbWydaWgaaWcbaGaeGOmaidabeaakiabdwgaLnaaCaaaleqabaGaeyOeI0Iaem4yam2aaSbaaWqaaiabiodaZaqabaWccqWG6bGEaaaaaa@3588@ where *c*_1_, *c*_2_, *c*_3 _are constants (taken to be 0.02, 0.15, and 5, respectively) and *z *is a random number (between 0 and 1). Then for each gene/TF pair, we assigned a random number *h*_*in *_(between 0 and 1). For parameter *e*, which determines how dense the synthetic TRN is, if *h*_*in*_*u*_*n *_<*e *we set *b*_*in *_= -1 (down regulation), if *e *≤ *h*_*in*_*u*_*n *_< 2*e*, we set *b*_*in *_= 1 (up regulation), assuming the probability of up and down regulation is the same. The *Q*_*in *_were allowed to change 20 fold and were generated randomly (in the logarithmic scale). TF activities were assumed to be random as well. Our synthetic examples with large TRNs show that, despite the simplicity of the FTF approach, the constructed TF activity profiles are reliable. To test the approach, one can compare the TF activities constructed and those used in the generation of synthetic expression data. For example, for a TRN that has the properties shown in Fig. 2, even when we eliminate 50% of the TRN to create a "preliminary TRN", 90% of the constructed TF activities have a Pearson correlation coefficient of at least 0.70 with the TF activities used to generate the synthetic expression data (when 20 or more microarray experimental conditions were used). Fig. 3 shows the dependence of the results on the number of experiments. This graph shows that, for practical reason, it is not feasible to recover the full network. Fig. 4a shows the effect of network structure on the results. As the network gets denser, the percentage of the network that can be recovered decreases. Fig. 4b illustrates the dependence of the percentage of recovery on the degree of incompleteness in the preliminary TRN. As anticipated, more complete preliminary TRNs allow a higher percentage of the unknown part of the network to be recovered using expression data. These results suggest that in a real world application such as *E. coli *(for which we have probably less than 40% of the TRN – based on the number of gene/TF interactions known and expected number of TFs), one can not expect to construct the full TRN using expression data alone, regardless of the number of expression datasets available.

### Multi-method TRND integration

Each of the above individual methods provides a score for each gene/TF interaction. The statistical significance of the score is assessed by the ratio of the probability of that score in the training set to that in the random set. For a given method we determine a score *R *for each gene/TF interaction as above. An experimentally-verified TRN of *E*. *coli *[14] is used as the training set to determine ftrk
 MathType@MTEF@5@5@+=feaafiart1ev1aaatCvAUfKttLearuWrP9MDH5MBPbIqV92AaeXatLxBI9gBaebbnrfifHhDYfgasaacH8akY=wiFfYdH8Gipec8Eeeu0xXdbba9frFj0=OqFfea0dXdd9vqai=hGuQ8kuc9pgc9s8qqaq=dirpe0xb9q8qiLsFr0=vr0=vr0dc8meaabaqaciaacaGaaeqabaqabeGadaaakeaacqWGMbGzdaqhaaWcbaGaemiDaqNaemOCaihabaGaem4AaSgaaaaa@326B@(*R*), the fraction of the known interactions in the training set in each of a number of intervals of *R* for methodology k, similarly frandk
 MathType@MTEF@5@5@+=feaafiart1ev1aaatCvAUfKttLearuWrP9MDH5MBPbIqV92AaeXatLxBI9gBaebbnrfifHhDYfgasaacH8akY=wiFfYdH8Gipec8Eeeu0xXdbba9frFj0=OqFfea0dXdd9vqai=hGuQ8kuc9pgc9s8qqaq=dirpe0xb9q8qiLsFr0=vr0=vr0dc8meaabaqaciaacaGaaeqabaqabeGadaaakeaacqWGMbGzdaqhaaWcbaGaemOCaiNaemyyaeMaemOBa4MaemizaqgabaGaem4AaSgaaaaa@34FB@(*R*) is obtained for randomly chosen gene/TF interactions for methodology *k*. If ftrk
 MathType@MTEF@5@5@+=feaafiart1ev1aaatCvAUfKttLearuWrP9MDH5MBPbIqV92AaeXatLxBI9gBaebbnrfifHhDYfgasaacH8akY=wiFfYdH8Gipec8Eeeu0xXdbba9frFj0=OqFfea0dXdd9vqai=hGuQ8kuc9pgc9s8qqaq=dirpe0xb9q8qiLsFr0=vr0=vr0dc8meaabaqaciaacaGaaeqabaqabeGadaaakeaacqWGMbGzdaqhaaWcbaGaemiDaqNaemOCaihabaGaem4AaSgaaaaa@326B@(*R*)/frandk
 MathType@MTEF@5@5@+=feaafiart1ev1aaatCvAUfKttLearuWrP9MDH5MBPbIqV92AaeXatLxBI9gBaebbnrfifHhDYfgasaacH8akY=wiFfYdH8Gipec8Eeeu0xXdbba9frFj0=OqFfea0dXdd9vqai=hGuQ8kuc9pgc9s8qqaq=dirpe0xb9q8qiLsFr0=vr0=vr0dc8meaabaqaciaacaGaaeqabaqabeGadaaakeaacqWGMbGzdaqhaaWcbaGaemOCaiNaemyyaeMaemOBa4MaemizaqgabaGaem4AaSgaaaaa@34FB@(*R*) >> 1, an interaction with a score *R *for a given method is highly likely to be correct. These Bayesian ratios are computed for each method and gene/TF interaction. The sum of the log_10 _of these ratios is taken to be the multi-method confidence measure *K*_*in*_:

Kin=∑k=1Nmethwklog⁡10(ftrk(Rink)frandk(Rink))     (4)
 MathType@MTEF@5@5@+=feaafiart1ev1aaatCvAUfKttLearuWrP9MDH5MBPbIqV92AaeXatLxBI9gBaebbnrfifHhDYfgasaacH8akY=wiFfYdH8Gipec8Eeeu0xXdbba9frFj0=OqFfea0dXdd9vqai=hGuQ8kuc9pgc9s8qqaq=dirpe0xb9q8qiLsFr0=vr0=vr0dc8meaabaqaciaacaGaaeqabaqabeGadaaakeaacqWGlbWsdaWgaaWcbaGaemyAaKMaemOBa4gabeaakiabg2da9maaqahabaGaem4DaC3aaSbaaSqaaiabdUgaRbqabaGccyGGSbaBcqGGVbWBcqGGNbWzdaWgaaWcbaGaeGymaeJaeGimaadabeaakmaabmaabaWaaSaaaeaacqWGMbGzdaqhaaWcbaGaemiDaqNaemOCaihabaGaem4AaSgaaOGaeiikaGIaemOuai1aa0baaSqaaiabdMgaPjabd6gaUbqaaiabdUgaRbaakiabcMcaPaqaaiabdAgaMnaaDaaaleaacqWGYbGCcqWGHbqycqWGUbGBcqWGKbazaeaacqWGRbWAaaGccqGGOaakcqWGsbGudaqhaaWcbaGaemyAaKMaemOBa4gabaGaem4AaSgaaOGaeiykaKcaaaGaayjkaiaawMcaaaWcbaGaem4AaSMaeyypa0JaeGymaedabaGaemOta40aaSbaaWqaaiabd2gaTjabdwgaLjabdsha0jabdIgaObqabaaaniabggHiLdGccaWLjaGaaCzcamaabmaabaGaeGinaqdacaGLOaGaayzkaaaaaa@6960@

where *w*_*k *_is a weighting factor, *N*_*meth *_is the number of TRN construction methodologies, Rink
 MathType@MTEF@5@5@+=feaafiart1ev1aaatCvAUfKttLearuWrP9MDH5MBPbIqV92AaeXatLxBI9gBaebbnrfifHhDYfgasaacH8akY=wiFfYdH8Gipec8Eeeu0xXdbba9frFj0=OqFfea0dXdd9vqai=hGuQ8kuc9pgc9s8qqaq=dirpe0xb9q8qiLsFr0=vr0=vr0dc8meaabaqaciaacaGaaeqabaqabeGadaaakeaacqWGsbGudaqhaaWcbaGaemyAaKMaemOBa4gabaGaem4AaSgaaaaa@3225@ is the score for TF *n *and gene *i *using methodology *k*, ftrk
 MathType@MTEF@5@5@+=feaafiart1ev1aaatCvAUfKttLearuWrP9MDH5MBPbIqV92AaeXatLxBI9gBaebbnrfifHhDYfgasaacH8akY=wiFfYdH8Gipec8Eeeu0xXdbba9frFj0=OqFfea0dXdd9vqai=hGuQ8kuc9pgc9s8qqaq=dirpe0xb9q8qiLsFr0=vr0=vr0dc8meaabaqaciaacaGaaeqabaqabeGadaaakeaacqWGMbGzdaqhaaWcbaGaemiDaqNaemOCaihabaGaem4AaSgaaaaa@326B@ and frandk
 MathType@MTEF@5@5@+=feaafiart1ev1aaatCvAUfKttLearuWrP9MDH5MBPbIqV92AaeXatLxBI9gBaebbnrfifHhDYfgasaacH8akY=wiFfYdH8Gipec8Eeeu0xXdbba9frFj0=OqFfea0dXdd9vqai=hGuQ8kuc9pgc9s8qqaq=dirpe0xb9q8qiLsFr0=vr0=vr0dc8meaabaqaciaacaGaaeqabaqabeGadaaakeaacqWGMbGzdaqhaaWcbaGaemOCaiNaemyyaeMaemOBa4MaemizaqgabaGaem4AaSgaaaaa@34FB@ are the probability distributions for the training set and random set, respectively. If a methodology fails to have a prediction for a gene-TF pair, it is excluded in the above calculation. The weighting factors are taken to be unity in this study.

## Results

**Figure 5:**
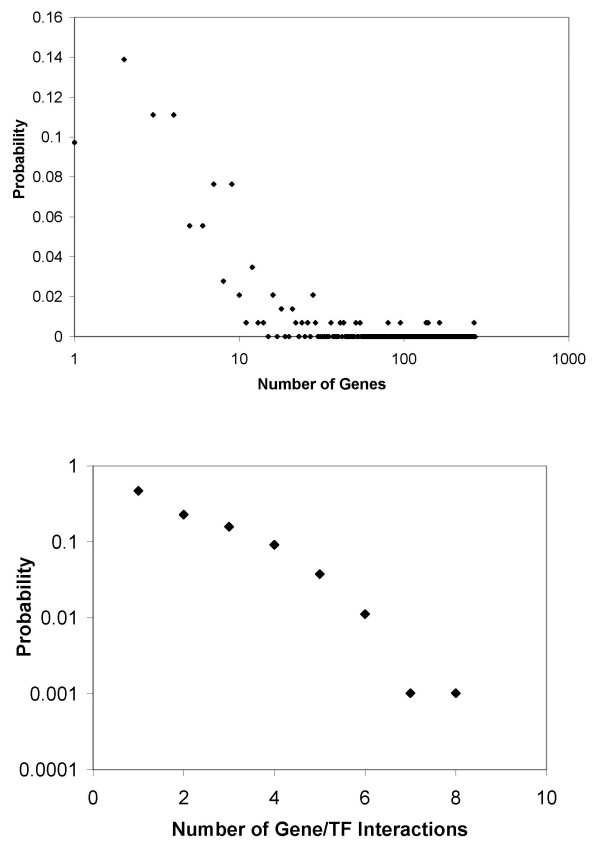
a) Probability distribution for the number of genes regulated by a given TF, b) probability distribution for the number of gene/TF interactions per gene. These graphs are based on the preliminary TRN taken from [14].

Our methodology requires a preliminary TRN which is used as the training set in all three methodologies presented below. We gathered this training set from EcoCyc [14]. EcoCyc describes *E. coli *operons, promoters, TFs, and TF binding sites. The database describes the mechanisms of transcriptional regulation of *E. coli *genes, and contains the most complete description of the genetic network of any organism. EcoCyc and RegulonDB [36] are curated to ensure that their data content is the same. The preliminary TRN used in this study included 984 genes, 144 TFs, and 2007 gene/TF interactions. Out of 2007 gene/TF interactions, 1124 were up regulation, 766 were down regulation, 5 were uncertain, and 112 were dual regulation (both up/down). Basic properties of the preliminary *E. coli *TRN are illustrated in Fig. 5.

**Figure 6:**
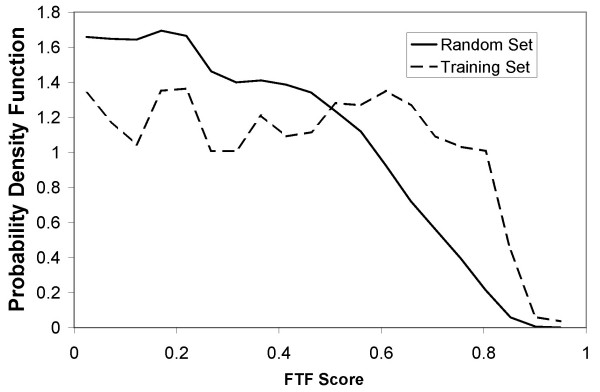
Probability distribution of FTF similarity scores of the training set (dashed) with respect the random set (solid). x-axis refers to FTF similarity score while y-axis refers to its probability distribution. A comparison with Fig. 1 (diamond markers) shows that our approach is superior to the gene-gene correlation approach.

We applied the FTF methodology to *E. coli *using expression datasets obtained from the NIH omnibus service: GSE7 (physiological and genetic changes that affect tryptophan metabolism), GSE8 (chromosomal replication forks in synchronized cells) and GSE9 (UV exposure). These 65 sets were chosen as the experiments were performed on the same platform. One single run of FTF on a PC (Xeon 2.4 GHz) takes about 15 minutes and requires 700 MB memory. The probability distributions for the absolute value of the Pearson correlation coefficient between the constructed TF activities (using equation 2) and expression data are shown in Fig. 6 for both the training and random sets. A comparison of Fig. 6 and Fig. 1 shows that by constructing TF activities using a preliminary TRN, we significantly increase the amount of information extracted from expression data.

**Figure 7:**
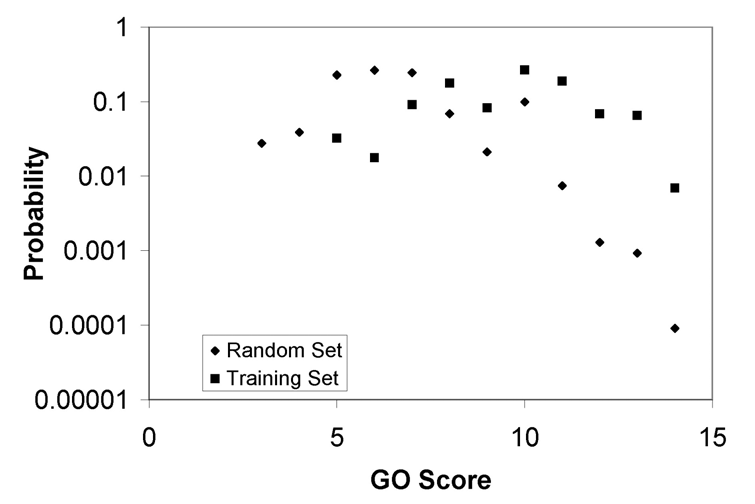
Comparison of the probability distributions of GO similarity scores of the training set (square markers) and the random set (diamond markers). The training set consists of all known *E. coli *gene/TF interactions for those genes with GO terms assigned. The random set consists of all possible gene/TF interactions for those genes with GO terms assigned. It is seen that higher GO similarity score implies higher likelihood of a gene/TF interaction, particularly when the GO similarity score is larger than 8.

Using the biological process ontology developed by the Gene Ontology Consortium, we calculated GO similarity scores. We then calculated gene/TF scores using the approach described in From Gene-Gene Scores to Gene-TF Scores Section. Fig. 7 shows the probability distributions for the training (gene/TF interactions in the preliminary TRN) and complete (all possible gene/TF interactions) sets. The significant variation between the training and random sets provides evidence that the likelihood for a gene pair to be regulated in the same manner increases with the similarity of their GO description. A comparison of Fig. 7 and Fig. 2 of Wu et al. (2005) shows that our approach is more successful in distinguishing the training and random sets (Note that [20] included pathway data in their training set whereas we only used the *E. coli *TRN).

**Figure 8:**
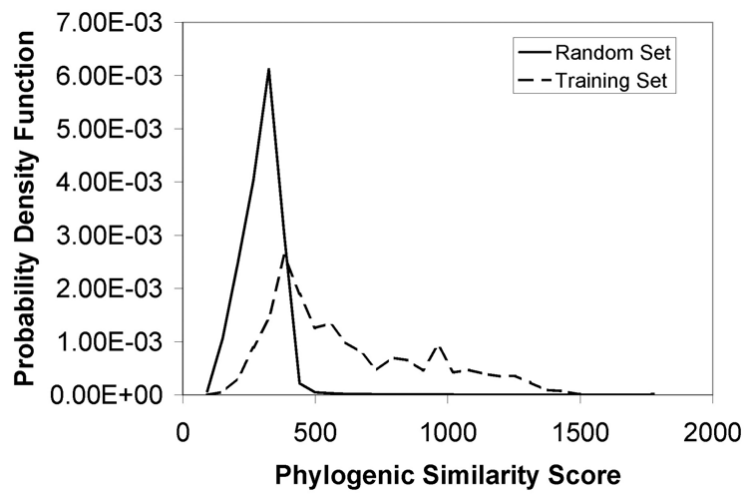
Comparison of the probability distributions of Phylogenic Similarity scores of the training set (dashed) and the random set (solid). x-axis refers to Phylogenic Similarity Score while y-axis refers to its probability distribution. The training set is based on all known gene/TF interactions from [14]. The random set consists of all possible gene/TF interactions. It is seen that higher score implies higher likelihood of a gene/TF interaction, particularly when the similarity score is greater than 500.

We extended the number of genomes used in the phylogenic similarity analysis from 134 to 229 and used the *E. coli *TRN as the training set in contrast to the gene-gene pair training set suggested by [20]. Fig. 8 shows the probability distributions for the training (gene/TF interactions in the preliminary TRN) and complete (all possible gene/TF interactions) sets. Phylogenic similarity outperforms the GO and FTF methodologies. As in the case for GO similarity, the results are better than those obtained earlier (Fig. 4 of [20]) due to the gene-TF versus the gene-gene based approach.

**Figure 9:**
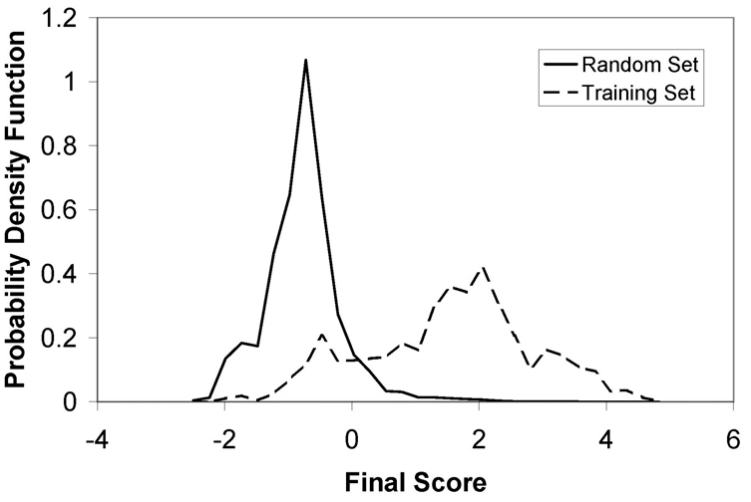
Probability distribution of combined scores for the training set (dashed) and the random set (solid). The training set is based on all known gene/TF interactions from [14]. The random set consists of all possible gene/TF interactions. It is seen that higher combined score implies higher likelihood of a gene/TF interaction.

The probability distributions of the integrated confidence score for the training and complete gene/TF sets are shown in Fig. 9. We applied a threshold of 1.3 to this score to find the most likely gene/TF interactions. To facilitate the use of our results by the research community, they are posted at [37] where users can view/download the results and perform search queries. As our procedure is automated, when new information and microarray or other data become available, the entire procedure can be readily repeated.

To provide an objective measure of deviations between two probability distributions, we calculated the chi-square scores for GO, phylogenic, and FTF analysis as well as the final integrated probability distributions (Figs. 6, 7, 8, 9). We created 4 bins for all distributions and calculated the number of gene/TF scores in each bin. Note that a chi-square score of 16.27 gives a p-value of 0.001 for a system with three degrees of freedom (number of bins minus one). We found the chi-square scores to be 49667 (phylogenic similarity), 13005 (GO), 579 (FTF), and 79584 (integrated). These scores indicate and GO and phylogenic similarity measures provide better predictions than expression analysis. Higher chi-square score for the integrated probability distributions justifies the integration scheme. A cross examination of scores from different methodologies has shown that if a gene/TF interaction scores high for one of the three methodologies, this doesn't imply that the remaining two methods will support this prediction. For example, out of the 1000 highest phylogenic similarity scores, only 48 and 3 of them were found in the top 1000 GO and FTF scores.

**Figure 10:**
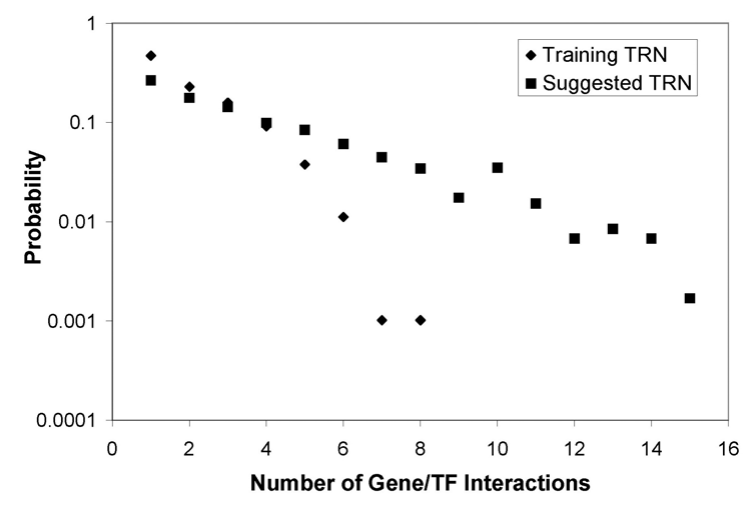
Probability distributions for the number of gene/TF interactions per gene. Although the suggested TRN is denser, the overall shape of the probability distribution remains the same.

The suggested TRN includes 3694 new gene/TF interactions. If the training TRN is a random sampling of the actual TRN, then, for a sufficiently large training TRN, it is expected to exhibit the basic functional properties of the actual TRN. The suggested TRN is denser than the training TRN. However, as illustrated in Fig. 10, probability distributions for the number of gene/TF interactions per gene for both the training and suggested TRNs show a high degree of similarity. Clearly, our training set is vastly incomplete. Not only we don't have any regulatory information for over 3,000 genes, but we likely know only a fraction of the number of TFs regulating those 984 genes for which at least one regulating TF is known. Therefore, the true *E. coli *TRN is likely to be denser, as predicted here.

After we performed the calculations we found 206 more gene/TF interactions in the RegulonDB and EcoCyc databases that were not included in the training set. 44 out of 206 regulatory interactions were predicted by our methodology. Out of 44 interactions, the nature of regulation was correctly predicted for 33 of them. Regulation type couldn't be obtained for 7 interactions. Regulation nature was incorrectly predicted for the remaining 4 interactions (Table 2). We obtained the p-value for predicting at least 44 out of 206 gene/TF interactions to be less than 1.0e-50 (expected proportion = 3.5e-04, number observed = 44, sample size = 3694).

**Table 2:** Out of 206 gene/TF interactions found in the RegulonDB (Salgado et al. 2004) and EcoCyc databases, 44 scored higher than the imposed threshold.

	TF	Gene	Final Score	predicted sign	actual sign
1	ArcA-Phosphorylated	yfiD	1.670768591	up	up
2	ArgR-L-arginine	argG	2.624262246	down	down
3	CRP-cAMP	ugpQ	2.085693237	up	up
4	CRP-cAMP	ugpC	1.438960585	up	up
5	CRP-cAMP	ugpB	1.527292985	up	up
6	CRP-cAMP	rhaB	2.909109432	up	up
7	CRP-cAMP	cytR	2.119101207	up	up
8	CRP-cAMP	fis	1.887330412	up/down	up/down
9	FhlA-Formate	hyfA	2.509458668	up	up
10	Fis	relA	1.595459732	up	up
11	GntR	idnK	1.591861262	down	down
12	Lrp	livJ	1.380451883	down	down
13	MalT-Maltotriose-ATP	malZ	1.44543234	up	up
14	NarL-Phosphorylated	fdhF	2.059069186	up	up
15	NtrC-Phosphorylated	astC	2.256990592	up	up
16	ArgR-L-arginine	astC	1.505034	down	up
17	CRP-cAMP	nagE	1.501987378	up/down	up
18	CRP-cAMP	rpoS	2.319114034	up/down	down
19	OmpR-Phosphorylated	nmpC	1.572242248	up	down
20	ArcA-Phosphorylated	aceE	1.998375233	down	down
21	ArcA-Phosphorylated	appC	2.066734924	up	up
22	CRP-cAMP	entC	2.93996178	up	up
23	CRP-cAMP	fepA	3.821927836	up	up
24	CRP-cAMP	fumA	3.496049117	up	up
25	CRP-cAMP	gadB	2.197333426	down	down
26	CRP-cAMP	galP	2.37334941	up	up
27	CRP-cAMP	gapA	1.811986697	up	up
28	CRP-cAMP	ompF	1.315706561	up	up
29	FruR	acnA	1.633652931	up	up
30	FruR	glk	2.112996214	down	down
31	GadE	gadB	1.973757857	up	up
32	Hns	gadB	1.78680429	down	down
33	LexA	uvrC	1.590391147	down	down
34	MetJ-S-adenosylmethionine	metE	3.264214502	down	down
35	MetJ-S-adenosylmethionine	metR	1.611151228	down	down
36	MetR-Homocysteine	metE	3.45265202	up	up
37	PhoP-Phosphorylated	rstA	1.6475787	up	up
38	CRP-cAMP	prpR	3.147648347	up/down	up
39	Fnr	fdhF	1.985483486	up/down	up
40	CRP-cAMP	nagE	1.501987378	up/down	up
41	NarP-Phosphorylated	fdhFp	1.645007039	up/down	up
42	NarP-Phosphorylated	fdnG	2.778075473	up/down	down
43	Fnr	dcuS	1.359810967	down	up
44	Fnr	purM	1.427076396	up	down

The p-value (using binary distribution) is found to be less than 1.0e-50.

We also used the gene expression data (described above in the microarray analysis section) to further test the suggested TRN as follows. We obtained approximate TF activities for both the training and suggested TRNs. Then, for each gene we calculated the linear correlation coefficient between the expression data and the sum of TF activity profiles (accounting separately up versus down regulation). Higher scores indicate better consistency between expression data and TRN. The average scores for the training and suggested TRNs were calculated to be 0.47 and 0.54, respectively, showing an improvement in the overall consistency of the TRN with gene expression profiles.

## Conclusion

We believe our results on *E. coli *demonstrate the viability of the multi-method approach for bacteria. The focus on gene/TF interactions rather than the gene/gene interaction approach apparently is a key to the approach and also yields more detailed information on the nature of the TRN. The Bayesian framework provides the objective interaction methodology.

The multi-method integration scheme straightforwardly generalizes to other techniques; thus we are presently adding promoter analysis and protein-protein interaction modules to the integrated scoring. We hope this type of computational analysis will guide experimental studies and accelerate research in the discovery of TRNs. We are applying the methodology to other bacteria of interest, notably *Geobacter sulfurreducens *and *Bacillus anthracis*.

## List of abbreviations

FTF Fast transcription factor analyzer

GO Gene ontology

NCA Network component analysis

TF Transcription factor

TRN Transcriptional regulatory network

TRND Transcriptional regulatory network discovery

## Competing interests

The author(s) declare that they have no competing interests.

## Authors' contributions

JS calculated the phylogenic similarity measure and contributed to the manuscript. KT designed the research idea, developed FTF, integrated and interpreted the results, and wrote the manuscript. AAH calculated the GO scores. FS, MT and LE prepared the web interface for the results. PO was involved in developing the research idea and editing the manuscript. All authors read and approved the final manuscript.
